# Intimate Partner Violence among Women with Disabilities in Uganda

**DOI:** 10.3390/ijerph16060947

**Published:** 2019-03-16

**Authors:** Anne Valentine, Ilhom Akobirshoev, Monika Mitra

**Affiliations:** The Lurie Institute for Disability Policy, The Heller School for Social Policy and Management, Brandeis University, Waltham, MA 02453, USA; ilhom@brandeis.edu (I.A.); mmitra@brandeis.edu (M.M.)

**Keywords:** intimate partner violence, disability, maternal, reproductive health, low- and middle-income country

## Abstract

Violence against women with disabilities is pervasive, yet a paucity of research examines intimate partner violence (IPV) experienced by women with disabilities in low- and middle-income countries. The purpose of this study is to document the prevalence and consequences of IPV exposure among Ugandan women with disabilities. Cross sectional data from the 2011 and 2016 Uganda Demographic and Health Surveys (UDHS) were used to study married and/or partnered women aged 15–49 who answered specific questions about lifetime intimate partner violence (*N* = 8592). Univariate and multivariate logistic regression models were used to investigate the relationship between disability, IPV, and indicators of maternal and child health. Compared to women without disabilities, women with disabilities were more likely to experience lifetime physical violence (odds ratio (OR) 1.4, *p* < 0.01), sexual violence (OR = 1.7, *p* < 0.01), and emotional abuse (1.4, *p* < 0.01) after controlling for sociodemographic and household characteristics. Study findings suggest that women with disabilities in Uganda may experience increased risk for IPV compared to women without disabilities, with concomitant risks to their health and the survival of their infants. Further research examining the prevalence and correlates of IPV in low- and middle-income countries is needed to address the needs and rights of women with disabilities.

## 1. Introduction

Violence against women is a pervasive, global public health problem [1]. Estimates suggest that 30–35% of ever-partnered women aged 15 years and older have experienced physical and/or sexual intimate partner violence (IPV) in their lifetime [1,2]. IPV is defined as physical, sexual, or emotional harm by a current or former spouse or partner [3], although research to date has primarily assessed the impact of physical and/or sexual violence by a partner. There is considerable heterogeneity globally, in both prevalence estimates of IPV and in the extent to which spousal abuse is considered acceptable and/or permissible [4,5,6], suggesting that contextual factors are an important determinant of violence against women [7,8].

Countries across sub-Saharan Africa experience very high rates of IPV and gender-based violence [2,9]. In 2016, 56% of ever-married Ugandan women reported IPV, with marked variations in prevalence estimates by geographic region and between rural and urban areas [10]. Between 2011 and 2016, the percentage of ever-married Ugandan women who reported experiencing physical, sexual, or emotional abuse by a current or former spouse decreased slightly, however, lifetime prevalence estimates remain high [11].

Women exposed to IPV experience a range of short- and long-term adverse physical and psychological harm including increased risk of unwanted pregnancy, pregnancy loss, sexually transmitted infection, HIV (Human Immunodeficiency Virus), posttraumatic stress disorder, depression, suicidality, and physical disability [2,12,13,14]. Several factors are associated with a woman’s risk of experiencing IPV including: young age, low education, unemployment of male partner, disparity between partners’ education, marital discord, societal acceptance of violence, child maltreatment, witnessing violence as a child, alcohol use, and gender attitudes that support the marginalization of women [15,16,17].

Research conducted in high-income countries suggests that disability is both a risk marker and a consequence of IPV in women. Women abused by an intimate partner are significantly more likely to report a disability or chronic condition that impacts their ability to carry out activities of daily living [18]; in low- and middle-income countries IPV is a leading cause of morbidity and mortality in reproductive aged women [19]. Prevalence estimates of physical and sexual violence experienced by disabled women are either similar to, or higher than, those reported by non-disabled women [20,21,22,23,24,25]. However, some scholars maintain that accurate prevalence rates do not exist given the scant attention paid to disability-related abuse in assessing IPV among women with disabilities [25].

Notably, there is research suggesting that the nature of victimization and abuse experienced by women with disabilities may be distinct from that experienced by women without disabilities. Women with disabilities are particularly at risk for severe violence [20,26], for sexual assault [27,28], and disability-related neglect [29,30]. In comparison to their male counterparts, women with disabilities are more likely to live in conditions characterized by poverty and isolation, increasing the likelihood that they experience violence without recourse [20,27]. Further, compared to non-disabled women, the duration of exposure to violence may be longer among disabled women [31] and disability-specific abuse is common [25].

The risk of IPV in women with disabilities is attributed to a number of complex contextual and social factors. Increased dependency on others for care, physical vulnerability, social isolation, and lack of economic independence are posited to make women with disabilities more vulnerable [27,31,32,33,34]; however, the devaluation of all persons with disabilities and the particular marginalization of sexuality in women with disabilities are important drivers of vulnerability as well. Consequently, some researchers maintain that violence against women with disabilities encompasses both disability-based and gender-based violence [35,36,37].

Despite the overwhelming evidence on the magnitude of violence against people with disabilities, there is a paucity of research on IPV among women with disabilities, particularly in low-income countries [30]. A majority of research to date has been conducted in the United States and the United Kingdom. A 2012 systematic review and meta-analysis of research examining violence against adults with disabilities included a single study from a middle-income county; no studies from low-income countries met the review’s inclusion criteria [30]. However, a few smaller studies are cited in the review [38,39], underscoring the need for rigorous research on the prevalence, correlates, and consequences of IPV among women with disabilities in low- and middle-income countries. To address these research gaps, the present study used national population-based data from the Uganda Demographic and Health Surveys (UDHS) to document the prevalence and health-related consequences of IPV among Ugandan women with disabilities.

## 2. Materials and Methods

### 2.1. Data and Sample

The study utilizes cross sectional data from the 2011 and 2016 Uganda Demographic and Health Surveys (UDHS) [10,11]. The UDHS provides up-to-date estimates of key demographic, socioeconomic, and health indicators in Uganda including sexual and reproductive health in adults, infant and maternal mortality, nutritional status, malaria, disability status, and intimate partner violence. The UDHS employed a multistage cluster sample survey design. In the first stage, enumeration areas (EAs) in urban and rural areas were selected. In the second stage, a random sample of approximately 30 households from each EA was selected for the survey. Detailed information about survey design and sampling method can be found elsewhere [10,11].

The UDHS data are nationally representative for women 15–59 years of age. A total of 27,180 women were interviewed in 2011 and 2016. Of these, 10,036 ever-married women and/or women cohabitating with a partner during the period of the survey were randomly selected to complete the IPV module. To ensure confidentiality, specially trained female interviewers conducted face-to-face interviews and were instructed to skip the module entirely and/or end the interview if a private setting was not available. Our analytic sample consists of 8592 women who were currently married/cohabitating with a partner and responded to the IPV module. We excluded 1444 women who were formerly married/cohabitating with a partner (i.e., widowed, divorced).

#### 2.1.1. Disability Measure

The main explanatory variable was disability, ascertained from responses to the Washington Group Short Set of Questions on Disability [40]. Disability status was assessed based on difficulties an individual may experience engaging in activities due to a problem(s) related to: (1) seeing; (2) hearing; (3) communicating; (4) remembering; (5) walking; and (6) washing or dressing. Responses were categorized on a continuum of no difficultly; some difficulty; a lot of difficulty; and cannot do at all. We employed the Washington Group on Disability Statistics’ analytical guidelines to create a dichotomous disability status indicator using the 2011 and 2016 UDHS data [41]. Women who reported “a lot of difficulty” or “cannot function at all” to any of the functional areas were classified as having a disability; women who reported “none” or “some difficulty” were classified as not having a disability.

#### 2.1.2. Intimate Partner Violence Measure

The main response variable was exposure to IPV including physical violence, sexual violence, and emotional violence perpetrated by a current or former husband/partner. Current married or cohabitating women were asked about experiences of IPV perpetrated by a husband or partner ever/in the past 12 months using an abbreviated version of the Conflict Tactics Scale [42]. Questions related to specific acts of violence such as being physically threatened or hurt, forced into sexual intercourse, or humiliated or insulted, were categorized into three domains denoting acts of physical, sexual, or emotional violence [43]. Those answering yes to any of the questions relating to physical, sexual, or emotional violence were classified as having experienced IPV. Respondents with negative response to all questions relating to physical, sexual, or emotional violence were classified as not experiencing IPV. Women exposed to multiple forms of IPV may have worse outcomes [44]; therefore, we created mutually exclusive categories for each of the three categories of violence as well as a combined indicator of IPV exposure.

### 2.2. Reproductive and Child Health Measures

In addition, the association between disability, IPV, and indicators of maternal and child health were examined, including: (a) use of modern contraceptive methods, (b) pregnancy loss/termination, (c) receipt of antenatal care during first trimester, (d) delivery at a health facility, and (e) infant mortality under 5 years of age. Use of modern contraceptive methods was based on response to a general inquiry about contraceptive use. Women who reported contraceptive use were asked about which method they used. Respondent’s use of either female sterilization, injectable contraceptive, intrauterine device(IUD), pill, implant, condom/female condom, diaphragm, or foam/jelly were classified as using a modern contraceptive method [45]. Ever terminated pregnancy was a binary indicator of a pregnancy loss resulting from a miscarriage, abortion, or stillbirth. Receipt of antenatal care during first trimester, delivery at a health center, and infant mortality under five years of age were all dichotomous variables. The maternal and child health outcomes among women without disabilities who reported no exposure to IPV (−/−) were compared to: (1) women without disabilities who reported exposure to IPV (−/+); (2) women with disabilities who reported no exposure to IPV (+/−); and (3) women with disabilities who reported exposure to IPV (+/+).

### 2.3. Covariates

Covariates used in the analysis were all among currently married/cohabiting women including: age (<25 years, 25–34 years, 35+ years), education (no education, primary, secondary, higher), union status (married, living together), age at marriage (<20 years, 20+ years), number of living children (0, 1, 2, 3, 4, or more), and occupation (not working, agricultural occupation, nonagricultural occupation). Household characteristics included household wealth quintile (lowest, second, third, fourth, highest) and place of residence (urban, rural, Kampala).

### 2.4. Data Analysis

Sociodemographic and household characteristics were compared among women with and without disabilities. Proportions were reported for categorical variables, with a Chi-square test used to test significance. Means and standard deviations were reported for continuous variables and significance was tested using t-tests. We used univariate and multivariate logistic regressions to investigate the relationship between disability status and various types of IPV. Because a number of covariates in our models had missing values, we conducted chained multiple imputation for our analyses, consistent with best practices [46,47]. All estimations in the descriptive and multivariate analysis corrected for the complex survey design of the UDHS. We performed all analyses using Stata, version 15 MP (StataCorp, College Station, TX, USA) [48]; a *p*-value of <0.05 was the accepted level of significance.

This work does not meet federal definitions of research; therefore, the project does not fall under the jurisdiction of the authors’ university Institutional Review Board in 2018.

## 3. Results

### 3.1. Women’s Characteristics

Table 1 describes the sample and provides bivariate contrasts in sociodemographic and household characteristics between Ugandan women with and without disabilities. In comparison to women without disabilities, women with disabilities were more likely to be older, have less education, and have more children. Other characteristics between women with and without disabilities, including union status, age at marriage, place of residence, and household wealth were insignificant.

### 3.2. Experience of Physical, Sexual, and Emotional Intimate Partner Violence

Nearly two-thirds (64%) of Ugandan women with disabilities reported ever experiencing physical, sexual, or emotional IPV, compared to slightly more than half (55%) of women without disabilities, as shown in Table 2. Higher percentages of women with disabilities experienced physical violence (49% vs. 39%), sexual violence (35% vs. 22%), and emotional violence (51% vs. 39%) during their lifetime, than their peers without disabilities. These associations remained after controlling for sociodemographic and household characteristics. Namely, compared to women without disabilities, women with disabilities had approximately 1.4 times higher odds (95% CI: 1.01–1.82, *p* < 0.05) of experiencing physical violence, 1.7 times higher odds (95% CI: 1.23–2.34, *p* < 0.01) of experiencing of sexual violence, and 1.4 times higher odds (95% CI: 1.09–1.91, *p* < 0.01) of experiencing emotional violence. Similarly, women with disabilities were at a higher risk for specific acts of violence, as shown in Table 2. For example, the odds of being physically forced to have sex were almost two times higher for women with disabilities compared to non-disabled women (1.67, 95% CI: 1.22–2.29, *p* < 0.01). Notably, no significant difference between women’s exposure to IPV by disability status was observed when we used the IPV composite measure, adjusting for sociodemographic and household characteristics.

### 3.3. Association between Intimate Partner Violence, Disability Status, and Health Outcomes

Table 3 reports unadjusted and adjusted odds ratios from bivariate and multivariable analyses for the associations between health outcomes and IPV by disability status. Adjusting for all covariates, we found that compared to women without disabilities with no exposure to IPV (−/−), women without disabilities with exposure to IPV (−/+) had 1.3 times higher odds (95% CI: 1.13–1.51, *p* < 0.01) of pregnancy loss or termination and 1.2 higher odds (95% CI: 1.02–1.43, *p* < 0.05) of delivery at a health facility. However, disabled women with no exposure to IPV (+/−) had even higher odds, 1.7 (95% CI: 1.06–2.68, *p* < 0.05) of pregnancy loss or termination and there was no significant difference in the likelihood of delivery at a health facility compared to the reference group. Compared to women without disabilities with no exposure to IPV (−/−), women with disabilities with exposure to IPV (+/+) had nearly twice the odds (OR = 1.95, 95% CI: 1.32–2.89, *p* < 0.01) of losing a child under five years of age.

## 4. Discussion

This secondary analysis of UDHS data is, to our knowledge, the first to examine the prevalence of IPV among women with disabilities in Uganda and the associated maternal and child health effects of co-occurring disability and exposure to lifetime intimate partner violence. Depending on the type of violence exposure, the adjusted odds ratios of ever experiencing IPV in women with disabilities ranges from 1.3 (physical violence) to 1.7 (sexual violence). Within each category of violence exposure, the type of indicator markedly influences the risks observed; the odds of being threatened with a knife, gun, or other weapon, for example, are more than two times higher among women with disabilities compared to women without disabilities. Given that IPV prevalence among all Ugandan women is markedly higher than the average rate reported globally (35%), our study findings suggest an even higher risk for IPV among married Ugandan women with disabilities compared to their non-disabled peers.

Consistent with previous research in higher income countries [20,21,22,23,24,25], this study documents the heightened vulnerability to physical, sexual, and emotional abuse experienced by married/partnered Ugandan women with disabilities. Notably, compared to non-disabled women, women with disabilities in Uganda experience nearly twice the risk of one or more indicators of sexual violence (i.e., being physically forced to have sexual intercourse and/or forced to perform other unwanted sexual acts). We suspect that the prevalence of sexual violence, as well as physical violence and emotional abuse, experienced by women with disabilities is apt to be considerably higher than the rates reported here, as research shows that women with disabilities are less likely to report violence and also less likely to be believed if they report abuse [49,50]. Additional personal and systems level factors including fear, stigma, dependence on perpetrators, attitudinal and structural barriers to information and supports often pose additional barriers to disclosure [25,50,51]. Though these barriers are likely common among non-disabled women, it is important that future research examines the additional and disability-specific barriers to preventing and reporting abuse.

For women with disabilities, examining the mechanisms by which disability and gender-based factors increase the risk for violence is essential to the development of interventions and policies that prevent and/or mitigate the risks and associated consequences of IPV. Little is known about the underlying norms related to violence among women with disabilities. Plausible explanations for the heightened association between IPV and disability status in Uganda include the social acceptance of violence and the role of male controlling behavior and power relations within intimate relationships with women with disabilities. There are several unique risk factors for women with disabilities that may increase their vulnerability to abuse including: dependence on the intimate partner for essential care; a lack of transportation or inaccessible transportation; pervasive stereotypes that characterize women with disabilities as weak or helpless; and non-existent or limited social services and supports for women with disabilities who experience abuse [36,52].

Although we did not examine male controlling behavior in this study, it may be that consistent with results from a multi-country examination of IPV and risk of HIV infection in sub-Saharan Africa, male controlling behavior is a risk factor for physical and sexual violence that is distinct from situational couple violence, which seems to be relatively common within married/cohabitating partners in many sub-Saharan African countries but is not characterized by a pervasive effort to dominate or exert control over a partner [13,53]. A 2015 study examining the association between male controlling behavior and intimate partner sexual violence in Uganda found that predictors of women’s empowerment did little to mitigate the risk of sexual violence, suggesting that interventions to prevent sexual violence ought to explicitly focus on men’s behavior and a seemingly pervasive tolerance of sexual violence within marriage [54,55]. Clearly there is an urgent need to understand the social norms related to violence and the role of control in intimate relationships among women with disabilities in Uganda. Further, given the high prevalence of emotional abuse reported by women with disabilities in Uganda, its relationship to impairment specific abuse (e.g., withholding/breaking assistive devices, withholding medications, refusing to assist with toileting, as needed) is an area of inquiry that deserves further investigation.

Consistent with other studies in low- and middle-income countries, our findings support significant associations between IPV and pregnancy loss/termination and infant mortality [56,57,58]. Many of the associations observed however, should be interpreted with caution as we are unable to disentangle the risk for pregnancy loss/termination among women with disabilities from the additive and/or independent adverse effects of IPV exposure in this analysis. Earlier studies have demonstrated that women who are deaf or hard of hearing, women with intellectual and/or developmental disabilities, and women who are at risk for disability are at greater risk for pregnancy complications and adverse birth outcomes [59,60,61,62,63]. Although this research was conducted in the United States and Canada, these associations merit further study in low- and middle-income countries, particularly among women residing in rural areas and among women with different disabilities—accounting for the severity of disability—as the correlates and consequences of IPV may be distinct [7,12]. Certainly, access to reproductive and child health services is likely very limited in some areas of Uganda [39], although difficulties in accessing reproductive health services among women with disabilities has been reported in urban areas as well [64].

This study contributes to an emerging body of research examining associations between IPV and disability among women in low-income countries using nationally representative data and robust, rigorous methods [37,58]. To date, this type of research has not been feasible. The UDHS’ early adoption of the Washington Group Short Set of Questions on Disability provides some of the first population-based data on disability in sub-Saharan Africa and constitutes an important step toward full implementation of the UNCRPD (Convention on the Rights of Persons with Disabilities) [65]. Certainly, more research is needed to examine the context, etiology, and consequences of IPV and to identify strategies to prevent violence against women with disabilities in Uganda. Uganda is lauded among sub-Saharan Africa countries for its progressive approach to advocating for the rights of individuals with disabilities [66]. However, evidence suggests that significant gaps remain with respect to its disability-related policies and practices [64,66]. Further, violence against women remains a pervasive problem [6,67]. Research highlighting the nature and consequences of IPV among women with disabilities may lead to policies and intervention efforts that mitigate the risks and consequences of IPV exposure among women with disabilities, their children, and families. We hope that this study stimulates interest in the inclusion of the needs of women with disabilities in policy and practice efforts to prevent IPV and to address the Sustainable Development Goals of gender equality and the elimination of gender-based violence among women with disabilities in the Global South [68,69].

### Limitations

There are several limitations to this study that are worth noting. First, the UDHS does not provide information about the severity, duration, onset, and cause of disability—all of which may limit the sensitivity and accuracy of the data provided. Second, the cross-sectional nature of the data also limits the extent to which we can infer temporality or causal associations between IPV and disability. Limited research to date allows for the examination of whether disability occurred before or after exposure to IPV [30]. Longitudinal studies are needed to further examine the sequence of disability and lifetime violence exposure among married women in Uganda. Further, because formerly married or cohabitating women were excluded from the analysis, information about abuse that occurs after separation was not included [17]. Third, as is typical with many secondary analyses, the study was limited by the presence of missing data. Missing data may be increased given the sensitive nature of the data examined; both intimate partner violence and pregnancy loss/termination may be under-reported. Multiple imputations by chained equations, consistent with best practices [46,47], were used to impute values for variables with missing data. All analyses were performed using the developer-provided sampling weights, a commonly accepted approach. Fourth, per the Washington Group on Disability Statistics’ analytical guidelines, we created a dichotomous disability status indictor; this may make the disability measure less reliable. Finally, the data were all self-reported and, as such, the findings were subject to potential recall and social desirability bias. Despite these limitations, this work is highly relevant to researchers, policymakers, and non-governmental organizations working across various sectors to prevent IPV and address the needs and rights of women with disabilities.

## 5. Conclusions

The current study revealed high rates of IPV experienced by women with disabilities in Uganda, with significant risks to their well-being, as well as the health and survival of their infants. The findings underscore the need for a body of research examining the prevalence and correlates of IPV in low- and middle-income countries among married/cohabitating women with disabilities.

## Figures and Tables

**Table 1:** Sample characteristics.

Characteristic	With Disability	No Disability	*p*-Value ^a^
	*n* = 299	*n* = 8293	
Women’s characteristics			
Maternal age			
<25 years	13.6%	31.1%	<0.001
25–34 years	37.4%	42.1%	0.814
35+ years	49.0%	26.8%	<0.001
Maternal age, Mean (SD)	34.4(0.5)	29.4(0.1)	
Education			
No Education	18.1%	12.8%	0.022
Primary	64.9%	60.3%	0.189
Secondary	14.3%	20.5%	0.030
Higher	2.6%	6.4%	0.017
Union status			
Living together	52.6%	49.3%	0.355
Married	47.4%	50.7%	0.355
Age at marriage/cohabitation			
20+ years	27.2%	28.8%	0.617
<20 years	72.8%	71.2%	0.617
Number of living children			
0	5.0%	6.5%	0.391
1	7.4%	13.6%	0.003
2	8.5%	17.7%	<0.001
3	14.3%	16.0%	0.509
4 or more	64.8%	46.3%	<0.001
Employment status			
Unemployed	15.2%	18.3%	0.284
Employed	84.8%	81.7%	0.284
Household characteristics			
Wealth quintile			
Lowest	20.6%	20.5%	0.959
Second	21.8%	21.1%	0.801
Third	22.8%	19.6%	0.237
Fourth	20.6%	18.4%	0.435
Highest	14.1%	20.4%	0.019
Place of residence			
Urban	13.5%	15.8%	0.352
Rural	82.4%	79.9%	0.355
Kampala	4.1%	4.3%	0.872

Source: Uganda Demographic and Health Surveys, 2011 and 2016. Notes: ^a^ *p*-values for differences, *χ*^2^-test or *t*-test.

**Table 2:** Associations between disability status and experience of specific acts of violence among women with and without disabilities, aged 15–49, Uganda: percentages, unadjusted and adjusted odds ratios.

Outcomes	With Disability *n* = 299	No Disability ^a^ *n* = 8288	Unadjusted		Model 1 ^b^		Model 2 ^c^	
	%	%	OR	95% CI	OR	95% CI	OR	95% CI
Experience any intimate partner violence	**63.5**	**54.7**	1.45 **	**(1.08–1.96)**	1.29	(0.94–1.76)	1.27	(0.93–1.74)
Experience any physical spousal violence	**48.9**	**38.5**	1.53 ***	**(1.15–2.02)**	1.37 **	**(1.02–1.85)**	1.35 **	**(1.01–1.82)**
Ever push you, shook you or throw something at you	28.7	17.7	1.87 ***	**(1.37–2.55)**	1.67 ***	**(1.21–2.31)**	1.64 ***	**(1.19–2.28)**
Ever slap you	42.6	33.0	1.51 ***	**(1.12–2.02)**	1.37 **	**(1.00–1.86)**	1.35 *	(0.99–1.82)
Ever twist your arm or pull your hair	19.2	10.8	1.97 ***	**(1.31–2.95)**	1.73 ***	**(1.14–2.62)**	1.72 ***	**(1.14–2.58)**
Ever punch you with his/her fist or something that could hurt you	20.9	14.3	1.58 ***	**(1.12–2.24)**	1.38 *	(0.97–1.97)	1.36 *	(0.95–1.96)
Ever kick you, drag you or beat you up	22.3	15.0	1.63 ***	**(1.13–2.36)**	1.46 *	(1.00–2.14)	1.44 *	(0.99–2.11)
Ever try to choke you or burn you on purpose	11.0	5.3	2.21 ***	**(1.26–3.87)**	1.87 **	**(1.06–3.31)**	1.85 **	**(1.05–3.25)**
Ever threaten or attack you with a knife, gun or other weapon	11.9	4.7	2.78 ***	**(1.68–4.59)**	2.23 ***	**(1.34–3.70)**	2.19 ***	**(1.32–3.64)**
Experience any spousal sexual violence	**34.6**	**22.4**	1.80 ***	**(1.31–2.45)**	1.73 ***	**(1.26–2.36)**	1.71 ***	**(1.26–2.34)**
Ever physically force you to have sexual intercourse with him/her even when you did not want to	32.4	21.1	1.80 ***	**(1.32–2.46)**	1.68 ***	**(1.23–2.31)**	1.67 ***	**(1.22–2.29)**
Ever force you to perform any other sexual acts you did not want to	11.6	5.1	1.80 ***	**(1.32–2.46)**	1.68 ***	**(1.23–2.31)**	1.67 ***	**(1.22–2.29)**
Experience any emotional spousal violence	**51.2**	**39.0**	1.64 ***	**(1.25–2.15)**	1.46 ***	**(1.10–1.94)**	1.44 **	**(1.09–1.91)**
Ever say or do something to humiliate you in front of others	29.5	19.5	1.72 ***	**(1.26–2.35)**	1.50 **	**(1.09–2.08)**	1.49 **	**(1.09–2.05)**
Ever threaten to hurt or harm you or someone close to you	26.0	17.9	1.61 ***	**(1.15–2.26)**	1.46 **	**(1.04–2.05)**	1.44 **	**(1.02–2.02)**
Ever insult you or make you feel bad about yourself	41.7	31.9	1.53 ***	**(1.16–2.03)**	1.37 **	**(1.02–1.83)**	1.35 **	**(1.01–1.81)**

Source: Uganda Demographic and Health Surveys, 2011, 2016, *** *p* < 0.01, ** *p* < 0.05, * *p* < 0.1. Notes: ^a^ women without disabilities served as the reference group in the unadjusted and multivariate regression analysis; ^b^ adjusted for maternal age, education, union status, age at marriage, number of living children, and occupation; ^c^ adjusted for maternal age, education, union status, age at marriage, number of living children, occupation, household wealth, and place of residence. Abbreviations: OR = odds ratio, CI = confidence interval.

**Table 3:** Odds ratios (OR) of select health outcomes among women with and without disabilities by experience of intimate partner violence, aged 15–49, Uganda: unadjusted and adjusted odds ratios.

Health Outcomes	No Disability no IPV (−/−)		No Disability IPV (−/+)		with Disability no IPV (+/−)		with Disability IPV (+/+)	
Currently use modern contraceptive method								
Unadjusted, OR (95% CI)	1.00	1.00	0.95	(0.83–1.08)	1.07	(0.68–1.68)	0.85	(0.58–1.24)
Adjusted, OR (95% CI)	1.00	1.00	0.92	(0.80–1.05)	0.98	(0.62–1.54)	0.87	(0.57–1.31)
Weighted number	8595							
Ever terminated pregnancy								
Unadjusted, OR (95% CI)	1.00	1.00	1.38 ***	**(1.21–1.58)**	2.06 ***	**(1.32–3.21)**	1.78 ***	**(1.23–2.58)**
Adjusted, OR (95% CI)	1.00	1.00	1.31 ***	**(1.13–1.51)**	1.69 **	**(1.06–2.68)**	1.39 *	(0.96–2.01)
Weighted number	8595							
First ANC visit within first trimester								
Unadjusted, OR (95% CI)	1.00	1.00	1.09	(0.95–1.26)	1.71 *	(0.92–3.18)	0.93	(0.59–1.48)
Adjusted, OR (95% CI)	1.00	1.00	1.02	(0.88–1.19)	1.68	(0.90–3.13)	0.84	(0.52–1.34)
Weighted number	6654							
Delivered at health facility								
Unadjusted, OR (95% CI)	1.00	1.00	1.58 ***	**(1.34–1.85)**	1.03	(0.55–1.94)	2.00 ***	**(1.20–3.34)**
Adjusted, OR (95% CI)	1.00	1.00	1.21 **	**(1.02–1.43)**	0.79	(0.41–1.52)	1.53	(0.90–2.61)
Weighted number	6692							
Had child who died <5 years								
Unadjusted, OR (95% CI)	1.00	1.00	1.37 ***	**(1.19–1.58)**	1.14	(0.68–1.89)	3.11 ***	**(2.14–4.52)**
Adjusted, OR (95% CI)	1.00	1.00	1.07	(0.91–1.25)	0.69	(0.39–1.23)	1.95 ***	**(1.32–2.89)**
Weighted number	8595							

Source: Uganda Demographic and Health Surveys, 2011, 2016, *** *p* < 0.01, ** *p* < 0.05, * *p* < 0.1. Notes: adjusted for maternal age, education, union status, age at marriage, number of living children, occupation, household wealth, and place of residence. Abbreviations: IPV = intimate partner violence; ANC = antenatal care; OR = odds ratio, CI = confidence interval.
